# Associations between postprandial triglyceride concentrations and sex, age, and body mass index: cross-sectional analyses from the Tromsø study 2015–2016

**DOI:** 10.3389/fnut.2023.1158383

**Published:** 2023-06-15

**Authors:** Mari Mikkelsen, Tom Wilsgaard, Sameline Grimsgaard, Laila A. Hopstock, Patrik Hansson

**Affiliations:** ^1^Department of Clinical Medicine, Clinical Nutrition Research Group, UiT The Arctic University of Norway, Tromsø, Norway; ^2^Department of Community Medicine, UiT The Arctic University of Norway, Tromsø, Norway; ^3^Department of Health and Care Sciences, UiT The Arctic University of Norway, Tromsø, Norway; ^4^Department of Food Studies, Nutrition and Dietetics, Uppsala University, Uppsala, Sweden

**Keywords:** lipids, non-fasting triglycerides, postprandial period, time since last meal, sex, age, body mass index, menopausal status

## Abstract

**Introduction:**

Elevated serum triglyceride concentrations increase the risk of developing atherosclerosis, the leading cause of cardiovascular disease. Postprandial triglyceride concentrations have shown to be a stronger predictor of cardiovascular disease compared to fasting triglycerides. It is therefore clinically relevant to study patterns of postprandial triglyceride concentrations in a general adult population.

**Aims:**

The aim of this cross-sectional analysis was to examine postprandial triglyceride concentrations in women and men, and the association with age, body mass index and menopausal status.

**Methods:**

Non-fasting blood samples from 20,963 women and men aged 40 years and older, attending the seventh survey of the Tromsø Study (2015–2016), were analyzed for postprandial triglyceride concentrations using descriptive statistics and linear regression models. Self-reported time since last meal before blood sampling was categorized into 1-h intervals with 7+ hours considered fasting.

**Results:**

Men had higher triglyceride concentrations compared to women. The pattern of postprandial triglyceride concentrations differed between the sexes. In women, the highest triglyceride concentration (19% higher compared to fasting level, *p* < 0.001) was found 3–4 h postprandially compared to 1–3 h in men (30% higher compared to fasting level, *p* < 0.001). In women, all subgroups of age and BMI had higher triglyceride concentrations than the reference group (age 40–49 years and BMI < 25 kg/m^2^), but no linear trend for age was observed. In men, triglyceride concentrations were inversely associated with age. Body mass index was positively associated with triglyceride concentration in both women (*p* < 0.001) and men (*p* < 0.001), although this association was somewhat modified by age in women. Postmenopausal women had significantly higher triglyceride concentrations compared to premenopausal women (*p* < 0.05).

**Conclusion:**

Postprandial triglyceride concentrations differed in groups of sex, age, body mass index, and menopausal status.

## 1. Introduction

Cardiovascular disease (CVD) is the most common cause of death in the world (1–3), and accounted for about one third of all deaths globally in 2019 (4). The main established risk factors for CVD are smoking, hypertension and dyslipidemia (5, 6). Evaluation of an individual’s lipid profile is usually performed with fasting blood samples (7). However, several studies show that postprandial triglyceride measurements can act as a stronger risk factor for CVD (8, 9), and particularly coronary artery disease (10), compared to fasting triglycerides. Additionally, recent updated guidelines from the European Society of Cardiology and the European Atherosclerosis Society suggest that postprandial blood lipids also can be used in screening and general risk estimation (11). There are practical advantages of using non-fasting samples, including improved patients acceptance (11), as they are not required to fast for 8 h or more prior to blood sampling. Additionally, triglyceride concentrations measured postprandially present information about the lipid metabolism after a meal, which is not detected in fasting blood samples (12). Metabolic abnormalities, such as hypertriglyceridemia, can cause triglyceride-rich lipoproteins and their remnants to circulate in the blood for a longer period than normal after a meal (12). This accumulation can facilitate the infiltration of small remnants into the arterial wall, contributing to the atherosclerotic process (12). As most individuals likely spend most of their day in a postprandial state, it is highly relevant to study this period. An expert panel statement, reviewing recent studies, recommends that postprandial triglyceride concentrations should be measured 4 h after a fat tolerance test, as to provide a good estimation of the postprandial triglyceride response (13). Four hours postprandially is a time point where individuals usually have their peak after normal meals (13), and triglyceride concentrations measured 2–4 h postprandially might also be stronger associated with CVD risk (9). However, as triglyceride concentrations normally remain elevated for 6–8 h in healthy individuals, it is of interest to examine concentrations measured hourly between <1 h to 7+ hours after meal consumption, and not only at the time of expected concentration peak. This allows comparison of patterns between stratified groups to identify potential differences that cannot be detected using one single measurement.

Several factors have been studied in relation to postprandial triglyceride concentrations. Studies show a sex difference in lipid profiles, including fasting concentrations (14) as well as the postprandial metabolism of triglycerides (15–17), where men generally have higher triglyceride concentrations compared to women (16). However, not all studies have found this sex difference to be statistically significant (15), but the majority of studies considering menopausal status show that premenopausal women have lower postprandial concentrations of triglycerides compared to men (16, 18, 19). Several factors associated with CVD are affected by estrogen, including blood lipids (20). During menopause, metabolic changes include an increase in postprandial triglyceride concentrations, as well as a reduced postprandial triglyceride clearance (21). Additionally, some CVD risk factors, for example diabetes mellitus, may affect the risk of CVD differently in men and women through a differential alteration of triglyceride concentrations (22), which highlights the relevance of studying men and women separately (22).

Most studies show a positive association between age and triglyceride concentrations, both measured fasting (23, 24) as well as postprandially (15, 25, 26). However, several studies investigating the association between age and postprandial triglyceride concentrations have methodological challenges, such as small sample sizes with less than 25 participants (15, 26), not studying men and women separately (26), or including women only (25). It is therefore useful to investigate postprandial triglyceride concentrations in a large study population where participants can be grouped by both sex and age.

Studies show that triglyceride concentrations are higher in obese individuals when compared to non-obese individuals (27, 28). However, the majority of these studies only compare two BMI groups instead of studying BMI as a continuous variable or by comparing several BMI groups. Additionally, while some studies set a cut-off between these two groups at BMI 30 kg/m^2^ (28), others may compare obese individuals (BMI ≥ 30 kg/m^2^) with normal weight individuals (BMI 18.5–24.9 kg/m^2^) (27), thus not including individuals with overweight. By including more than two BMI groups, one could investigate whether there is a difference in triglyceride concentrations between normal- and overweight individuals, as well as between normal weight and obese individuals.

The primary aim of this study was to investigate associations between postprandial triglyceride concentrations and sex, age, and body mass index (BMI). The secondary aim was to examine whether there is an association between menopausal status and postprandial triglyceride concentrations.

## 2. Materials and methods

### 2.1. Study population

The Tromsø Study (29, 30) is an ongoing population health study conducted in the municipality of Tromsø, Norway. It was initiated in 1974 and consists of seven repeated surveys (Tromsø1-Tromsø7, 1974–2016). Total birth cohorts and random samples of the inhabitants have been invited (65–79% attendance), and 45,473 women and men have participated in one or more surveys. Data collection comprises questionnaires, interviews, measurements, biological sampling, and clinical examinations.

### 2.2. Study sample

**FIGURE 1:**
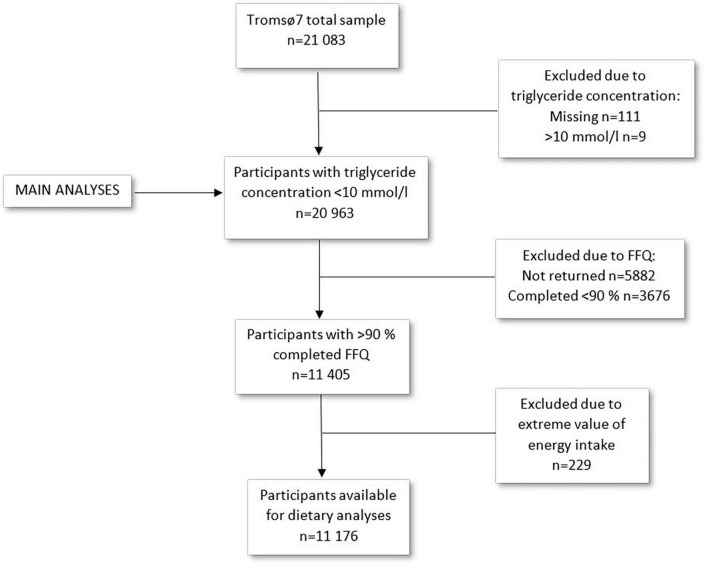
Flow chart of the study sample. The Tromsø Study 2015–2016.

This study includes data from the seventh survey of the Tromsø Study (Tromsø7) conducted in 2015–2016 (31). All inhabitants aged 40 years and older (*n* = 32,591) were invited to participate. A total of 21,083 women and men attended (65% attendance). Data collection included questionnaires including a food frequency questionnaire (FFQ), blood samples, measurements, and clinical examinations. Data collection was performed by trained personnel using standard methods. For the current study, participants with missing (*n* = 111) or extreme values (*n* = 9) of triglyceride concentration (>10 mmol/l) were excluded, leaving 20,963 participants for analysis (Figure 1). In total, 15,146 participants submitted the FFQ (72% of the total sample). In sub-group analyses including dietary factors, participants were excluded based on low (<90%) completeness of FFQ (*n* = 9,558) or extreme values of total energy intake (upper and lower 1%) (*n* = 229), in accordance with Lundblad et al. (32), leaving 11,176 participants for analyses including dietary factors (Figure 1).

### 2.3. Ethics and privacy

Data collection for Tromsø7 was approved by the Norwegian Data Protection Authority and the Regional Ethics Committee for Medical Research North (reference 2014/940). For the current study, data access was approved by the Tromsø Study Data- and Publication Committee upon application. All participants in Tromsø7 signed an informed consent.

### 2.4. Measurements

Non-fasting blood samples were drawn from an antecubital vein after a brief stasis, with the participant seated. Laboratory analyses were performed at the University Hospital of Northern Norway (NS-EN ISO 15189:2012) (33). Triglycerides were analyzed by enzymatic colorimetric methods with Cobas 8000 c702 (Roche Diagnostics, Mannheim, Germany). At blood sampling, participants were asked about time since last meal. This variable originally consisted of 10 intervals: <1 h, 1–1.59 h, 2–2.59 h, 3–3.59 h, 4–4.59 h, 5–5.59 h, 6–6.59 h, 7–7.59 h, 8–8.59 h, and 9+ hours. The last three time intervals were combined, and 7+ hours was considered fasting. Age was divided in intervals of 10 years (40–49 years, 50–59 years, 60–69 years, 70–79 years, and 80+ years). Age 40–49 years was set as reference group as this group was considered most homogeneous, least likely to be affected by disease. Weight and height were measured with light clothing and no shoes with a Jenix DS-102 scale (Dong Sahn Jenix, Seoul, Korea). BMI was calculated as weight divided by the square of the height (kg/m^2^) categorized based on the World Health Organization’s definition of under- and normal weight (<25 kg/m^2^), overweight (25–29.9 kg/m^2^), and obesity (≥30 kg/m^2^) (34). The under- and normal weight group was chosen as reference group. Additionally, the following covariates were included: smoking (Do you/did you smoke daily? Never, yes now, yes previously), recent alcohol consumption (When did you last drink alcohol? 0–2 days since, 3–6 days since, 7+ days since, never drank alcohol), recent physical exercise (When was your last physical exercise? 0–2 days since, 3–6 days since, 7+ days since), habitual physical activity [4 categories with increasing level of physical activity in accordance to Saltin and Grimby (35)], cholesterol lowering medication (Do you use or have used cholesterol lowering drugs? Never used, currently, previously not now), and education (What is the highest level of education you have completed? Primary/partly secondary education <10 years of schooling, upper secondary education ≥ 3 years, tertiary education short: college/university <4 years, tertiary education long: college/university ≥ 4 years). Smoking, use of lipid lowering drugs, recent alcohol intake, and recent physical activity were all recoded into binary variables. Smoking and medication use were dichotomized into current smokers/users and previous/never smokers/users. Recent physical exercise and recent alcohol intake were dichotomized into exercise/alcohol last 0–2 days (no/yes). The following dietary factors were included in a sub-group regression analysis: daily total energy intake in kilojoule (kJ), energy percentage (E%) of sugar and alcohol, and n-3 fatty acids supplements (in capsules per day).

In secondary analyses, two different definitions of menopause were used. First, women were divided based on an age cut-off at 50 years (50–59 years compared to 40–49 years). According to the literature, mean age for menopause is approximately 51–51.5 years (36, 37). With the 10-year age intervals in this dataset, the closest possible cut-off was 50 years, and as this was close to mean age for menopause, it was considered a reasonable cut-off for this secondary analysis. The group above 50 years had an upper limit of 59 years to include only women recently going through menopause, thereby avoiding selection bias in the older age groups due to disease. The second definition was based on self-reported information regarding menstruation status (Do you still menstruate? Yes/no) and reason for ended menstruation [If you do not have natural menstruation, why did it stop? It stopped by itself, uterus surgery, both ovaries were removed, hormonal intrauterine device, or other reason (e.g., radiation, chemotherapy)]. These two questions were combined to one variable defining women who still menstruated as premenopausal and those who reported menstruation stopped by itself as postmenopausal. Women reporting other reasons for ended menstruation were excluded.

### 2.5. Statistical analyses

*T*-test and chi-square test were used to examine sex differences in the study population. Multivariable linear regression models were used to assess the association between triglycerides as dependent variable and BMI (in three groups) and age (in five groups) as exposure variables in sex specific models. Assumptions for linear regression were evaluated through visual inspection of histograms of residuals, residual plots, and QQ-plots. Triglyceride concentration was log-transformed to achieve normal distribution. Two-way interactions between age and BMI, time since last meal and BMI, and time since last meal and age were assessed by adding cross product terms between indicator variables for each exposure group to models that included all main effects. Non-significant interactions were stepwise removed, leaving the interaction between age and BMI for the main analysis. In the main analysis, age and BMI were combined to one variable with 15 levels, and indicator variables for each combination were included in the model leaving those with BMI < 25 kg/m^2^ and age 40–49 years as reference group. In separate analyses, sex differences were investigated by testing the following interactions: sex by age, sex by BMI, and sex by time since last meal. All regression coefficients were back transformed so that all effects are interpreted as relative differences in triglyceride concentrations. The following covariates were adjusted for as possible confounders: physical activity, education, smoking, use of lipid lowering drugs, recent physical exercise, and recent alcohol intake.

In sub-group analyses including dietary factors, energy intake (kJ/d), sugar intake (E%), alcohol intake (E%), and supplements of n-3 fatty acids were included to assess whether these dietary factors affected the results.

The secondary analyses, investigating the association between menopausal status and postprandial triglyceride concentrations, were conducted with a similar model as described for the main analysis. In these analyses, the first model defined menopausal status based on an age cut-off at 50 years and the second model defined menopausal status based on self-reported data from the questionnaire. As the interaction between BMI and age was significant in women in the main analysis, this interaction was also tested for in the first secondary analysis where menopause was defined by age. This interaction was not significant and therefore not included in the model. The second secondary analysis, defining menopause based on menstruation status, did not include age. The interaction between BMI and menstruation was tested and as this interaction was significant, it was included in the model.

All analyses were cross-sectional as data material was collected from one survey. SPSS Statistics 28 was used for conducting statistical analyses and a *p*-value < 0.05 was considered statistically significant.

## 3. Results

### 3.1. Study sample characteristics

Figure 1 shows number of participants left after exclusions. A few additional participants were excluded from the regression models due to missing values for covariates included in the analyses. The numbers of included participants in the regression models are presented in the results from the respective analyses.

Table 1 shows characteristics of the study sample. There was a somewhat higher proportion of women aged 50–59 years and a somewhat higher proportion of men aged 60–69 years and 70–79 years. More women were normal weight (BMI < 25.0) and fewer women were overweight (BMI 25.0–29.9) compared to men. The proportions of women and men with obesity (BMI > 30.0) were more similar. More women had a low activity level, and more men had a moderate activity level. The difference between women and men with a sedentary or vigorous activity level was less prominent. There was also a minor difference in the distribution of educational level between men and women. There were more daily smokers among women compared to men, and a larger proportion of men were currently using lipid lowering drugs. Men had higher triglyceride concentrations compared to women.

**TABLE 1:** Characteristics of the study population.

		Men (*n* = 9,950)	Women (*n* = 11,013)	Total (*n* = 20,963)	*P*-value^1^
Age groups (years)					0.042
	40–49	30.5 (3,037)	30.5 (3,364)	30.5 (6,401)	
	50–59	27.8 (2,768)	29.3 (3,228)	28.6 (5,996)	
	60–69	25.0 (2,488)	24.2 (2,661)	24.6 (5,149)	
	70–79	13.2 (1,311)	12.3 (1,350)	12.7 (2,661)	
	80+	3.5 (346)	3.7 (410)	3.6 (756)	
BMI (kg/m^2^)					<0.001
	<25.0	23.7 (2,351)	39.8 (4,368)	32.1 (6,719)	
	25.0–29.9	50.8 (5,044)	37.4 (4,106)	43.8 (9,150)	
	≥30.0	25.5 (2,528)	22.8 (2,505)	24.1 (5,033)	
Physical activity level^2^					<0.001
	Sedentary	15.4 (1,492)	13.8 (1,458)	14.6 (2,950)	
	Low	50.4 (4,890)	65.0 (6,862)	58.0 (11,752)	
	Moderate	30.3 (2,939)	18.8 (1,989)	24.3 (4,928)	
	Vigorous	3.9 (380)	2.3 (248)	3.1 (628)	
Education					<0.001
	Primary	22.0 (2,155)	24.1 (2,603)	23.1 (4,758)	
	Upper secondary	30.5 (2,982)	25.4 (2,746)	27.8 (5,728)	
	Tertiary short	21.3 (2,084)	17.7 (1,910)	19.4 (3,994)	
	Tertiary long	26.1 (2,553)	32.9 (3,556)	29.7 (6,109)	
Recent alcohol intake	Consumed alcohol last 0–2 days	34.7 (3,445)	28.8 (3,166)	31.6 (6,611)	<0.001
Recent physical exercise	Physical exercise last 0–2 days	45.0 (4,461)	41.6 (4,563)	43.2 (9,024)	<0.001
Lipid lowering drugs	Current use	17.3 (1,696)	12.8 (1,382)	15.0 (3,078)	<0.001
Smoking	Daily smoking	13.2 (1,304)	14.5 (1,578)	13.9 (2,882)	0.009
Triglycerides	Median (IQR)	1.49 (1.10)	1.18 (0.81)	1.30 (0.95)	
Mean (SD)	1.71 (1.00)	1.35 (0.76)	1.52 (0.90)	<0.001

The Tromsø Study 2015–2016. After exclusion of participants with missing value for triglyceride concentration or triglycerides > 10 mmol/L. Numbers are percentages (number), median (interquartile range) or mean (standard deviation). BMI, body mass index (kg/m^2^); IQR, interquartile range; SD, standard deviation.

^1^*P*-values are difference between women and men tested by Student’s t-test for continuous variables and chi-square for categorical variables.

^2^Physical activity; self-reported leisure time physical activity in accordance to Saltin and Grimby (35).

### 3.2. Factors associated with triglyceride concentrations

#### 3.2.1. Sex differences

We observed significant interactions between sex and time since last meal (*p* < 0.001) and between sex and age (*p* < 0.001), meaning that the association between both time since last meal and triglycerides, as well as the association between age and triglycerides, were different in men and women. Men had in general higher triglyceride concentrations than women.

#### 3.2.2. Time since last meal

**FIGURE 2:**
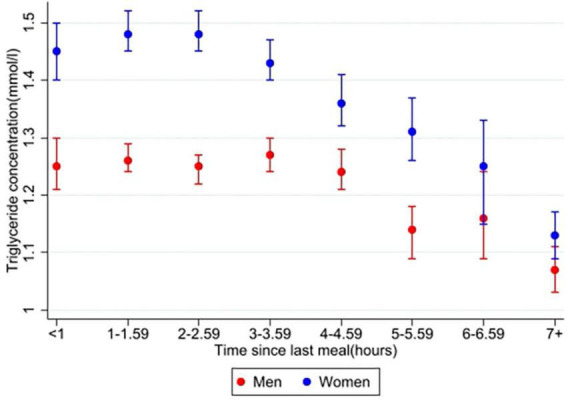
Estimated marginal means of triglyceride concentrations in men and women. The Tromsø Study 2015–2016. Values are adjusted for variables included in the main analysis (10,143 women and 9,583 men); time since last meal, age, BMI, physical activity, education (only women), smoking, use of lipid lowering drugs (only women), recent physical exercise, and recent alcohol intake. Error bars show 95% CI.

In the main analysis, time since last meal was significantly associated with triglyceride concentration in both men (*n* = 9,341) and women (*n* = 10,143) (overall *p* < 0.001 for both sexes). Relative differences for time, age and BMI, as well as for covariates, from the main analysis are presented in Supplementary Table 1. Figure 2 shows the triglyceride concentrations in men and women at specific time intervals after meal consumption, adjusted for covariates. Women had the highest triglyceride concentration 3–3.59 h after their last meal, with a 19% higher concentration compared to the fasting (7+ hours) value. Triglyceride concentration in women after 6–6.59 h also seemed to be higher than the previous time interval and the following time intervals. However, the difference between each time interval was not tested for. In men, the highest triglyceride concentrations were measured 1–1.59 and 2–2.59 h after meal consumption, both 31% higher than the fasting value. All triglyceride concentrations in the following time intervals were each lower than the previous.

#### 3.2.3. Age and body mass index

**FIGURE 3:**
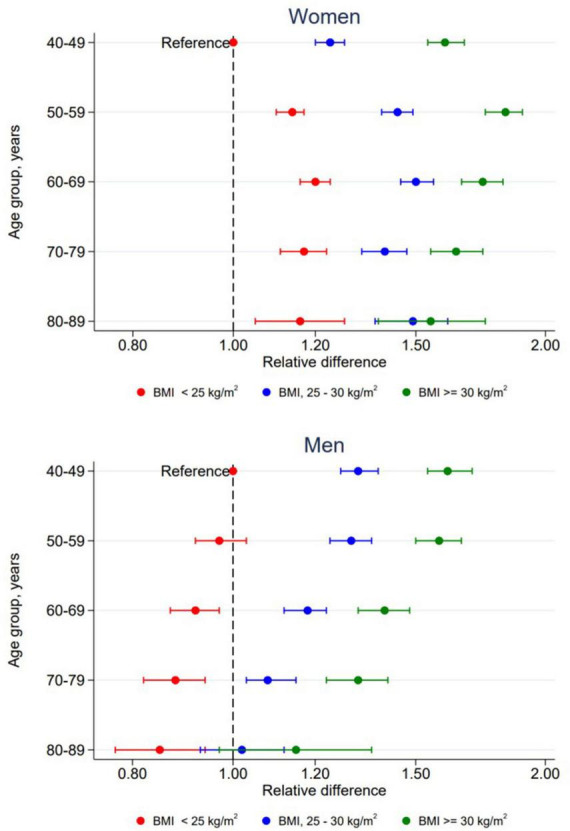
Relative difference in triglyceride concentrations according to combined levels of age and BMI by sex. The Tromsø Study 2015–2016. All relative differences are adjusted for time since last meal, physical activity, education, smoking, use of lipid lowering drugs, recent physical exercise and recent alcohol consumption with participants in age group 40–49 years with BMI < 25 kg/m^2^ as reference level. Error bars show 95% CI.

In women, both age and BMI were significantly associated with triglyceride concentration, overall *p* < 0.001. There was also a significant interaction between age and BMI in women, *p* < 0.001. Results from this interaction is plotted in Figure 3 and specific values are presented in Supplementary Table 1. All two-way combinations of age and BMI had higher triglyceride concentrations than the reference level (age 40–49 years and BMI < 25 kg/m^2^). Within each BMI group, no linear age trend was observed. Triglyceride concentrations were positively associated with BMI in all age groups, but the extend of the BMI-associated differences in triglyceride concentration varied between the age groups. In men, there was no significant interaction between age and BMI, *p* = 0.32. Triglyceride concentrations was positively associated with BMI in all age groups and inversely associated with age in all BMI groups.

#### 3.2.4. Dietary factors

Overall, including dietary factors (total energy intake, intakes of sugar, alcohol, and n-3 supplements) in the sub sample with valid dietary data had minor impact on the results. In women (*n* = 5,650), only intake of sugar and alcohol was significantly associated with triglyceride concentration, although the differences were small. Sugar intake was positively associated with triglyceride concentrations (+0.7% per one unit increase of sugar E%, *p* < 0.001) and alcohol intake was associated with lower triglyceride concentrations (−0.5% per one unit increase of alcohol E%, *p* = 0.015) in women. In men (*n* = 4,978), none of the dietary factors were significantly associated with triglyceride concentration.

#### 3.2.5. Menopause

Table 2 shows the association between two different definitions of menopause and triglyceride concentration. In the analysis defining menopause by age (*n* = 6,346), there was no significant interaction between menopause and BMI, *p* = 0.741. There was a significant difference in triglyceride concentration between pre- and postmenopausal women. Women in the age group 50–59 years had 14% higher triglyceride concentration compared to those in age group 40–49 years. When including dietary factors in a sub-group analysis (*n* = 3,480), women above 50 years had 16% higher triglyceride concentration compared to women under 50 years. The secondary analysis dividing women into two groups based on self-reported menstruation status (*n* = 8,034) also showed a significant difference in triglyceride concentration between the two groups. Postmenopausal women had 17, 16, and 5% higher triglyceride concentrations in normal weight, overweight, and obese groups, respectively, compared to premenopausal women. When including dietary factors in a sub-group analysis (*n* = 4,503), women who reported naturally ended menstruation showed 20, 17, and 7% higher triglyceride concentrations in normal weight, overweight, and obese groups, respectively, compared to the women still menstruating.

**TABLE 2:** Associations between menopausal status and triglyceride concentration.

Variable for menopausal status	RD^1^	95% CI^1^
**Age (years)**		
40–49 years (premenopausal)	1	Reference
50–59 years (postmenopausal)	1.14	[1.11, 1.16]
**Menstruation by BMI**		
BMI < 25 kg/m^2^		
Still menstruating	1	Reference
Menstruation stopped by itself	1.17	[1.14, 1.21]
**BMI 25–29.9 kg/m^2^**		
Still menstruating	1	Reference
Menstruation stopped by itself	1.16	[1.13, 1.21]
**BMI ≥ 30 kg/m^2^**		
Still menstruating	1	Reference
Menstruation stopped by itself	1.05	[1.01, 1.10]

The Tromsø Study 2015–2016. BMI, body mass index; RD, relative difference.

^1^Based on two models where one model defines menopause by menstruation status and the second model defines menopause by age (40–49 years defined as premenopausal and 50–59 years defined as postmenopausal, excluding women aged 60+ years). Values are adjusted for time since last meal, BMI, physical activity, education, smoking, lipid lowering drug use, recent exercise and recent alcohol consumption.

## 4. Discussion

In this study, postprandial triglyceride concentrations and associations with sex, age, BMI, and menopause were investigated in a Norwegian general population of adults and elderly. We found that time since last meal was significantly associated with triglyceride concentration in both men and women. Men had higher postprandial triglyceride concentrations, and their highest concentrations were measured earlier after meal consumption, compared to women. In women, all sub-groups of age and BMI had higher triglyceride concentrations compared to the reference group (40–49 years and BMI < 25 kg/m^2^), although no linear trend in age was seen. In men, triglyceride concentrations were inversely associated with age, independently of BMI, and BMI was positively associated with triglyceride concentration. Two different categorizations of menopausal status both showed higher triglyceride concentration in postmenopausal women compared to premenopausal.

Men had significantly higher concentrations of triglycerides compared to women. These findings are consistent with previous studies investigating sex differences in postprandial triglyceride responses (15, 18). The participants were not asked about additional information regarding their last meal, i.e., there is no information regarding the amount of food ingested or the composition of the meal, which can affect the triglyceride response (38). Given that men in general have a higher energy intake than women, there is a possibility that male participants ate larger meals (and thereby more fat) prior to blood sampling, leading to higher postprandial concentrations of triglycerides, compared to women. However, although meal size might affect the magnitude of the postprandial triglyceride response, it does not necessarily explain the different patterns seen in women and men.

The number of triglyceride peaks during the postprandial phase may vary between individuals, and studies have found responses with one, two, and three peaks (15, 39). An experimental study found two peaks (after 1 h and 6 h) in triglyceride concentration in women after given a carbohydrate rich meal (40), which is consistent with the results of the present study. The participants of our population study were free-living and were not exposed to a standardized high-fat test meal, as used in experimental studies investigating postprandial triglyceride concentrations. A high-fat test meal might not result in the same metabolic lipid response as participants’ normal meals during an average day (41). Therefore, the results of our study are interesting and may be of importance as they represent the participants’ triglyceride concentrations during a state in which they spend most of their time in everyday life. Studies show that a meal consumed 5 h prior to a test meal can induce an early peak in triglyceride concentration about an hour after meal consumption (42–44). Additionally, a second meal effect can alter triglyceride concentrations up to several hours after eating (19, 38). The participants in our study were not asked to fast before their last reported meal, as is common in experimental studies investigating postprandial triglyceride concentrations after a prepared test meal. With consecutive meals and in-between snacks during a day, it is likely that the measured triglyceride concentrations in the participants of our study are affected by other foods consumed prior in time to their last reported meal.

### 4.1. Age and body mass index

In women, all combinations of age and BMI had higher triglyceride concentrations compared to the reference group (40–49 years and BMI < 25 kg/m^2^), without any observed linear trend for age within each BMI group. This may indicate that the association is partly explained by menopause, which often occurs around the age of 51–52 years (36, 37). Additionally, this association was not seen in men, indicating that the association might be due to factors that differs between sexes. Several studies find a positive association between age and postprandial triglyceride concentrations (15, 26, 45). However, some of these studies have very few participants or do not study the effect of age in men and women separately. One study, investigating the effect of age on postprandial triglyceride concentrations in women, found a positive association, but only in premenopausal women, indicating an effect of metabolic changes due to the perimenopausal phase (25).

In men, triglyceride concentrations were inversely associated with age. Although most studies observe a positive and significant association between age and postprandial triglyceride concentration, there are also studies that do not find any association (46, 47). Inconsistent results may indicate that the results are confounded by other factors, as there are several factors that may influence the triglyceride concentration in the postprandial phase, for example physical activity and body composition (45, 46, 48). It is also possible that there is no association between age and postprandial triglyceride concentrations, or that the effect of age is small and therefore require a large study sample to uncover it. Our study included a large study sample and should therefore be able to uncover a potential association between age and postprandial triglyceride concentrations, given that the right confounding factors are included. Also, although with an age-range from 40 years and up, we cannot exclude the possibility of selection bias which may partly explain the inverse association. Few studies have investigated the effect of age on postprandial triglyceride concentrations in large samples stratified by sex. This might be due to demanding logistics of conducting a fat tolerance test (which is the most accurate way of studying postprandial triglycerides) with repeated blood sampling from a large group of participants. However, these types of studies are much needed and would be valuable to determine whether age is associated with postprandial triglyceride concentrations, independently of other factors.

BMI was significantly associated with triglyceride concentration in both men and women, where a higher BMI group was associated with a higher triglyceride concentration (although somewhat modified by age in women). These results are consistent with other findings, as postprandial triglyceride concentrations are shown to be higher in obese compared to non-obese individuals (28, 49). A positive association between BMI and postprandial triglyceride concentration is also seen when comparing normal weight to overweight, and obese young adults (45). The elevated concentrations of triglycerides observed in obese individuals may be partly explained by increased amounts of free fatty acids transported to the liver, which increases the production of very low-density lipoprotein (VLDL) particles (50). The VLDL particles compete with chylomicrons to be hydrolyzed by lipoprotein lipase (LPL), resulting in a slower clearance (50). Additionally, reduced LPL activity in skeletal muscles and decreased expression of LPL in adipose tissue will further slowdown the lipolysis of triglycerides from lipoproteins in individuals with obesity (50). Thus, the elevated postprandial triglyceride concentrations lead to a slower clearance, resulting in accumulation of VLDL- and chylomicron remnants in the arterial wall, initiating atherosclerosis (50).

### 4.2. Dietary factors

In women, only sugar- and alcohol intake showed significant associations with triglyceride concentrations. Sugar intake was positively associated with triglyceride concentrations in women. It has been shown that a diet rich in highly digestible carbohydrates can contribute to increased fasting triglyceride concentration through altered secretion, as well as postprandial clearance of triglyceride-rich lipoproteins from the blood (19). Additionally, the effect of sugar might depend on the amount, as well as type; while addition of glucose to a test meal has not shown a clear effect, both fructose and sucrose added to a test meal have been associated with an increase in postprandial triglyceride concentrations (19). Alcohol intake was inversely associated with triglyceride concentrations in women. Although several observational studies show a positive linear association between alcohol consumption and triglyceride concentration (51), a J-shaped curve has also been described (52, 53). A J-shaped relationship would not be detected in the present analyses as the regression models assume a linear relationship. However, a J-shaped relationship could explain the results in the present study, given that the majority of the participants reporting a regular alcohol intake have a light to moderate intake and thus lower triglyceride concentrations than those who do not drink, resulting in an inverse association. In men, none of the dietary factors were significantly associated with triglyceride concentration, which encourages further investigation.

Supplements of n-3 fatty acids were not significantly associated with triglyceride concentrations, which can seem unexpected, as a lowering effect of n-3 on triglyceride concentrations is reported in intervention studies (54), meta-analyses (55), and guidelines for the management of dyslipidaemias (11). Use of n-3 supplements (eicosapentaenoic acid and docosahexaenoic acid) can be recommended with the intention to lower triglyceride concentrations (11), thus, one could expect to see an association between n-3 supplementation and triglyceride concentration in the present study. One possible explanation for the lacking association could be the small variation in the use of supplement across various sub-groups, making it less likely to detect a potential association. Additionally, some participants might have started taking supplements due to altered triglyceride concentrations, which also could affect the results. The intake of fatty fish in the population could also affect the results but unfortunately this was not included in the data the authors accessed for the present study. However, the intake of fatty fish in Tromsø has been studied earlier. In one study, using data from Tromsø4 (56), it was observed an inverse trend between total intake of fish and triglyceride concentrations, as well as between the use of n-3 supplements and triglyceride concentrations, but no significant association between intake of fatty fish and triglyceride concentrations. Characteristics of the study population also showed a positive association between intake of fatty fish and the use of fish oil supplements. These analyses were adjusted for sex and age, but not for other covariates (56).

### 4.3. Menopause

When dividing women into pre- and postmenopausal groups based on an age cut-off, postmenopausal women had significantly higher triglyceride concentrations (14–16%) compared to premenopausal women. Similar results were seen when dividing women based on self-reported menstruation status (5–19% difference between pre- and postmenopausal women). These results are consistent with other studies investigating the effect of menopause on postprandial triglyceride concentrations (21, 25). Due to estrogen deficiency, several changes in the female body are observed during menopause and the following years. Weight gain and redistribution of body fat, accompanied by increased insulin resistance, elevated blood pressure, and altered plasma lipids are some of the observed changes related to increased risk of CVD in postmenopausal women (57). Change in body composition includes decreased lean body mass and increased total, as well as abdominal, body fat (58). Abdominal obesity is, as previously mentioned, associated with increased production of triglyceride-rich VLDL particles and reduced clearance of these lipoproteins from the blood (59). During menopause, women can develop a less favorable lipid profile, including elevated fasting and postprandial triglyceride concentrations, increased low-density lipoprotein (LDL)-cholesterol concentration, and decreased high-density lipoprotein (HDL)-cholesterol concentration (21, 60).

In the analysis defining menopause based on menstruation status, there was an interaction between menopausal status and BMI. In the normal and overweight groups, postmenopausal women had 18% and 17% higher triglyceride concentrations, respectively, compared to premenopausal women. In the obese group the difference was 5%. The main analysis showed a positive association between BMI and triglyceride concentration. It is therefore possible that the smaller difference seen in obese pre- and postmenopausal women, compared to normal- and overweight pre- and postmenopausal women, is partly due to obese women having already elevated triglyceride concentrations.

There is no gold standard consensus to define menopausal status in epidemiological studies, and the proportion of women being defined as pre- and postmenopausal can vary considerably across studies (61). In the present study, the difference in each definition included two samples: The first secondary analysis (with an age cut-off) excluded all women above 60 years, and the second analysis (based on menstruation status) excluded all women reporting unnatural reasons for ended menstruation. Different methods for defining menopause, as well as different sample sizes, could explain why a significant interaction between menopause and BMI was found in the menstruation status analysis but not in the model with an age cut-off.

### 4.4. Strengths and weaknesses

A strength of this study is the large study sample which made it possible to create all two-way combinations between age and BMI in sex specific models. The large sample allowed for comparison of postmenopausal women with premenopausal women aged 40 years and older, and to conduct analyses with dietary factors, although this required exclusion of several participants. Validated questionnaires were used for collecting data on diet (62), as well as habitual physical activity level (63). The results from this study represents the actual postprandial triglyceride concentrations in a general population, which can be considered clinically relevant and useful in future research.

As previously mentioned, unknown food intake earlier during the day of blood sampling may influence the triglyceride concentrations. However, having the participants to fast prior to a controlled test meal would have been logistically challenging and costly with the sample size of Tromsø7. Lack of a definition of a meal in the questionnaire asking participants about time since last meal may lead to measurement error as people may report different time since their last meal, based on their personal definition of a meal (any caloric intake vs. sitting down and eating a proper meal). Population studies are susceptible to selection bias which must be considered when interpreting the study results. No non-response analyses are available.

## 5. Conclusion

In this study, men had higher concentrations, and a different pattern, of postprandial triglycerides compared to women. In women, triglyceride concentrations were higher for all categories of higher age and BMI. In men, triglyceride concentrations were inversely associated with age and positively associated with BMI. Postmenopausal women had higher triglyceride concentrations compared to premenopausal women. Some uncertainties arise when studying postprandial triglyceride concentrations without a standardized test meal, including unknown meal composition, size, second meal effect, and the dependence on self-reported time since last meal. However, this study contributes with new clinically relevant information as it shows triglyceride concentrations representing the everyday life of individuals in a general population.

## Data availability statement

The datasets presented in this article are not readily available because the data analyzed in this study is subject to the following licenses/restrictions: The dataset is available upon application to the Tromsø Study. Requests to access the datasets should be directed to www.uit.no/research/tromsostudy.

## Ethics statement

The studies involving human participants were reviewed and approved by the Regional Ethics Committee for Medical Research North. The patients/participants provided their written informed consent to participate in this study.

## Author contributions

MM, LH, and PH designed the study. LH and SG contributed to the data collection. MM performed statistical analyses and wrote the first draft of the manuscript. TW provided statistical input. All authors contributed to the manuscript revision and read and approved the submitted version.
