# Supplementary material for: Associations between postprandial triglyceride concentrations and sex, age, and body mass index: cross-sectional analyses from the Tromsø study 2015–2016
Source: Front Nutr. 2023 Jun 15;10:1158383. doi: 10.3389/fnut.2023.1158383 (PMC10308115; doi:10.3389/fnut.2023.1158383)
Supplement: Supplementary file 1 [file Table_1.DOCX]

Supplementary Material

Supplementary Table 1. Parameter estimates for variables included in the main analysis. The Tromsø study 2015-2016.

|  | **Variable** | **Category** | **RD^1^** | **95% CI** | |
| --- | --- | --- | --- | --- | --- |
| **Women**  **(n=10 143)** | **Time since last meal (hours)** |  |  |  | |
|  |  | <1 | 1.17 | [1.12, 1.23] | |
|  |  | 1-1.59 | 1.18 | [1.14, 1.23] | |
|  |  | 2-2.59 | 1.17 | [1.12, 1.21] | |
|  |  | 3-3.59 | 1.19 | [1.14, 1,23] | |
|  |  | 4-4.59 | 1.16 | [1.11, 1.21] | |
|  |  | 5-5.59 | 1.06 | [1.01, 1.12] | |
|  |  | 6-6.59 | 1.09 | [1.01 1.17] | |
|  |  | 7+ | 1 | Reference | |
|  |  |  |  |  | |
|  | **BMI by age** |  |  |  | |
|  | **BMI <25 kg/m^2^** | **Age (years)** |  |  | |
|  |  | 40-49 | 1 | Reference | |
|  |  | 50-59 | 1.14 | [1.10, 1.17] | |
|  |  | 60-69 | 1.20 | [1.16, 1.24] | |
|  |  | 70-79 | 1.17 | [1.11, 1.23] | |
|  |  | 80 + | 1.16 | [1.05, 1.28] | |
|  | **BMI 25-29.9 kg/m^2^** | **Age (years)** |  |  | |
|  |  | 40-49 | 1.24 | [1.23, 1.28] | |
|  |  | 50-59 | 1.44 | [1.39, 1.49] | |
|  |  | 60-69 | 1.50 | [1.45, 1.56] | |
|  |  | 70-79 | 1.40 | [1.33, 1.47] | |
|  |  | 80 + | 1.49 | [1.37, 1.61] | |
|  | **BMI** ≥**30 kg/m^2^** | **Age (years)** |  |  | |
|  |  | 40-49 | 1.60 | [1.54, 1.67] | |
|  |  | 50-59 | 1.83 | [1.75, 1.90] | |
|  |  | 60-69 | 1.74 | [1.66, 1.82] | |
|  |  | 70-79 | 1.64 | [1.55, 1.74] | |
|  |  | 80 + | 1.55 | [1.38, 1.75] | |
|  |  |  |  |  | |
|  | **Physical activity^2^** |  | 0.95 | [0.94, 0.97] | |
|  |  |  |  |  | |
|  | **Recent physical exercise** | Physical exercise last 0-2 days | 0.93 | [0.91, 0.95] | |
|  |  |  |  |  | |
|  | **Recent alcohol intake** | Consumed alcohol last 0-2 days | 0.98 | [0.96, 0.99] | |
|  |  |  |  |  | |
|  | **Education** |  | 0.98 | [0.97, 0.99] | |
|  |  |  |  |  | |
|  | **Smoking** | Daily smoking | 1.16 | | [1.13, 1.19] |
|  |  |  |  | |  |
|  | **Lipid lowering drugs** | Current use | 1.08 | [1.05, 1,11] | |
|  |  |  |  |  | |
| **Men (n=9583)** | **Time since last meal (hours)** |  |  |  | |
|  |  | <1 | 1.28 | [1.22, 1.34] | |
|  |  | 1-1.59 | 1.31 | [1.26 1.36] | |
|  |  | 2-2.59 | 1.31 | [1.26, 1.36] | |
|  |  | 3-3.59 | 1.27 | [1.22, 1.32] | |
|  |  | 4-4.59 | 1.20 | [1.15, 1.25] | |
|  |  | 5-5.59 | 1.16 | [1.10, 1.22] | |
|  |  | 6-6.59 | 1.09 | [1.01, 1.18] | |
|  |  | 7+ | Reference | |  |
|  |  |  |  |  | |
|  | **BMI by age** |  |  |  | |
|  | **BMI <25 kg/m^2^** | **Age (years)** |  |  | |
|  |  | 40-49 | 1 | Reference | |
|  |  | 50-59 | 0.97 | [0.92, 1.03] | |
|  |  | 60-69 | 0.92 | [0.87, 0.97] | |
|  |  | 70-79 | 0.88 | [0.82, 0.94] | |
|  |  | 80 + | 0.85 | [0.77, 0.94] | |
|  | **BMI 25-29.9 kg/m^2^** | **Age (years)** |  |  | |
|  |  | 40-49 | 1.32 | [1.27, 1.38] | |
|  |  | 50-59 | 1.30 | [1.24, 1.36] | |
|  |  | 60-69 | 1.18 | [1.12, 1.23] | |
|  |  | 70-79 | 1.08 | [1.03, 1.15] | |
|  |  | 80 + | 1.02 | [0.93, 1.12] | |
|  | **BMI** ≥**30 kg/m^2^** | **Age (years)** |  |  | |
|  |  | 40-49 | 1.61 | [1.54, 1.70] | |
|  |  | 50-59 | 1.58 | [1.50, 1.66] | |
|  |  | 60-69 | 1.40 | [1.32, 1.48] | |
|  |  | 70-79 | 1.32 | [1.23, 1.41] | |
|  |  | 80 + | 1.15 | [0.97, 1.36] | |
|  |  |  |  |  | |
|  | **Physical activity level^2^** |  | 0.94 | [0.92, 0.95] | |
|  |  |  |  |  | |
|  | **Recent physical exercise** | Physical exercise last 0-2 days | 0.91 | [0.89, 0.93] | |
|  |  |  |  |  | |
|  | **Recent alcohol intake** | Consumed alcohol last 0-2 days | 0.98 | [0.96, 1,00] | |
|  |  |  |  |  | |
|  | **Smoking** | Daily smoking | 1.09 | [1.06, 1.12] | |

BMI; body mass index (kg/m^2^), RD; relative difference.

^1^Adjusted for time since last meal, BMI, age, physical activity, education, smoking, lipid lowering drug use, recent exercise and recent alcohol consumption.

^2^ Physical activity; self-reported leisure time physical activity in accordance to Saltin and Grimby (103)
